# Time to definitive diagnosis of breast cancer in Latina and non-Hispanic white women: the six cities study

**DOI:** 10.1186/2193-1801-2-84

**Published:** 2013-03-05

**Authors:** Amelie G Ramirez, Eliseo J Pérez-Stable, Gregory A Talavera, Frank J Penedo, J Emilio Carrillo, Maria E Fernandez, Edgar Muñoz, Dorothy Long Parma, Alan EC Holden, Sandra San Miguel de Majors, Anna Nápoles, Sheila F Castañeda, Kipling J Gallion

**Affiliations:** 1 Institute for Health Promotion Research, Department of Epidemiology and Biostatistics, The University of Texas Health Science Center at San Antonio, San Antonio, TX USA; 2 Division of General Internal Medicine, Medical Effectiveness Research Center for Diverse Populations, Department of Medicine, University of California, San Francisco, CA USA; 3 Institute for Behavioral and Community Health, Graduate School of Public Health, San Diego State University, San Diego, CA USA; 4 Department of Medical Social Sciences, Northwestern University, Chicago, IL USA; 5 Weill Cornell Medical College, Cornell University, New York, NY USA; 6 Center for Health Promotion and Prevention Research, University of Texas – Houston Health, Science Center School of Public Health, Houston, TX USA; 7 The National Latino Cancer Research Network, Institute for Health Promotion Research, Cancer Therapy & Research Center, The University of Texas Health Science Center at San Antonio, 7411 John Smith Drive, Suite 1000, San Antonio, TX 78230 USA

**Keywords:** Health disparities, Breast cancer screening, Definitive diagnosis, Latinas, Six cities study

## Abstract

Time delay after an abnormal screening mammogram may have a critical impact on tumor size, stage at diagnosis, treatment, prognosis, and survival of subsequent breast cancer. This study was undertaken to evaluate disparities between Latina and non-Hispanic white (NHW) women in time to definitive diagnosis of breast cancer after an abnormal screening mammogram, as well as factors contributing to such disparities.

As part of the activities of the National Cancer Institute (NCI)-funded *Redes En Acción* research network, clinical records of 186 Latinas and 74 NHWs who received abnormal screening mammogram results were reviewed to determine the time to obtain a definitive diagnosis. Data was obtained from participating clinics in six U.S. cities and included demographics, clinical history, and mammogram characteristics. Kaplan-Meier estimates and Cox proportional hazards models were used to test differences in median time to definitive diagnosis by ethnicity after adjusting for clinic site, demographics, and clinical characteristics.

Time-to-event analysis showed that Latinas took 2.2 times longer to reach 50% definitively diagnosed with breast cancer relative to NHWs, and three times longer to reach 80% diagnosed (*p=0.001*). Latinas’ median time to definitive diagnosis was 60 days compared to 27 for NHWs, a 59% gap in diagnosis rates (adjusted Hazard Ratio [aHR] = 1.59, 95% CI = 1.09, 2.31; *p=0.015*). BI-RADS-4/5 women’s diagnosis rate was more than twice that of BI-RADS-3 (aHR = 2.11, 95% CI = 1.18, 3.78; *p=0.011*).

Disparities in time between receipt of abnormal screening result and definitive diagnosis adversely affect Latinas compared to NHWs, and remain significant after adjusting for demographic and clinical variables. With cancer now the leading cause of mortality among Latinos, a greater need exists for ethnically and culturally appropriate interventions like patient navigation to facilitate Latinas’ successful entry into, and progression through, the cancer care system.

## Introduction

Despite notable progress in the overall health of Americans in general, disparities continue to persist in the burden of illness and death experienced by Hispanics/Latinos, as well as other ethnic groups, compared to the U.S. population as a whole (Agency for Healthcare Research and Quality 2008). With an estimated current population of more than 45 million, Latinos are one of the largest, youngest, and fastest-growing minority groups in the nation, representing about 15% of the total U.S. population (U.S. Census Bureau, American Community Survey 2006). It has been projected that by the year 2050, Latinos will represent a quarter of the population of the entire country (102.6 million) (U.S. Census Bureau 2004). Cancer recently superseded heart disease as the leading cause of morbidity and premature death in this minority group (Siegel et al. 2012), and breast cancer is the most frequently diagnosed cancer among Latinas (American Cancer Society 2009).

Breast cancer mortality has been declining steadily since 1990 by an average of 2.3% per year (Howe et al. 2006). However, while Latina women have lower breast cancer incidence (90.2 per 100,000) than do non-Hispanic white (NHW) or African American women (126.9 and 116.1 per 100,000, respectively), the decrease in incidence has been smaller (0.9% compared to 1.5% per year for NHWs). The disease continues to be the leading cause of cancer mortality among Latinas, with a five-year cause-specific survival rate 5% lower than NHW women (American Cancer Society 2009; Howe et al. 2006; American Cancer Society 2011a). Reduction in mortality is directly related to detection of early-stage breast cancer, because treatment is more effective and treatment options and survival rates are greater at that time (Vahabi 2003). There is strong evidence from clinical trials that regular screening mammography reduces mortality through early detection and treatment (Elmore et al. 2005; Feig 2005; U.S. Preventive Services Task Force 2002; Green & Taplin 2003; Knutson & Steiner 2007). Historically, Latina women have been less likely to utilize mammography services than NHW women and, consequently, more likely to be diagnosed at a more advanced stage of the disease, when fewer treatment options are available (Gorin et al. 2006). In 2010, Latina women aged 40 and older lagged behind both NHW and African American women (46.5% compared to 51%), in reporting they had a screening mammogram in the past year according to National Cancer Institute (NCI) Physician Data Query guidelines (PDQ Screening and Prevention Editorial Board 2012) and American Cancer Society (ACS) guidelines (American Cancer Society 2011b). This difference disappeared in two nationwide surveys using updated U.S. Preventive Services Task Force (USPSTF) recommendations of biennial screening for women aged 50–74 (U.S. Preventive Services Task Force, Screening for breast cancer 2009): in the National Health Interview Survey (NHIS), 69.7% of Latinas and 72.8% of NHWs aged 50–74 reported having a mammogram in the past two years (2012a), while the Behavioral Risk Factor Surveillance System (BRFSS) reported an equal proportion (75.4%) of Latinas and NHWs aged ≥ 40 screened (Miller et al. 2012). However, low-income Latinas and other ethnic minority women are more likely to delay or miss follow-up appointments (Ell et al. 2007; Guerra et al. 2005; Jones et al. 2005). These behaviors may result in poorer outcomes and higher mortality (American Cancer Society 2009; Blackman & Masi 2006; Newman & Martin 2007; Ramirez et al. 2000).

Many barriers contribute to Latinas’ lack of, or delay in, follow-up when breast abnormalities are first seen (Kaplan et al. 2005), as well as delay in treatment following diagnosis (Fedewa et al. 2011; Shavers & Brown 2002). Patients face the difficult challenge of understanding and navigating the highly complex nature of, and the structural barriers inherent in, the U.S. health care system, while dealing with the emotional and physical aspects of their health concerns and needs (Heckman et al. 2004). Barriers include knowledge and culturally-specific health beliefs leading to mistrust of the healthcare system and clinical research trials; competing health, family, and work responsibilities (Steinberg et al. 2006); reduced access to care; lack of insurance and social support; cost; language issues; lack of transportation and child care; psychological distress; poor physician-patient communication; and system inefficiencies (Gorin et al. 2006; Ell et al. 2007). Patients often become frustrated or discouraged and choose to discontinue the breast care they need. Although the majority of women lost to follow-up after an abnormal mammogram will eventually return to the system, they will most likely present with a more advanced stage of the disease.

This study was undertaken to evaluate disparities in time to definitive diagnosis after an abnormal screening mammogram between Latina and NHW women in six U.S. cities participating in the NCI-funded *Redes En Acción* research network (the Six City Study) (Ramirez et al. 2012), as well as factors contributing to such disparities. We hypothesized that: 1) Latinas experience significant delays in obtaining definitive diagnosis of breast cancer compared with NHWs; and 2) these delays are a result of a combination of patient- and clinic-associated factors.

## Materials and methods

### Study design and procedures

Between October 2006 and December 2007, after obtaining Institutional Review Board (IRB) approvals at UTHSCSA and each participating study site, we conducted a retrospective cohort study at six different sites: San Francisco, San Diego, Miami, New York, and Harlingen and San Antonio, Texas. Each site had high concentrations of Latinos. NCI-funded project *Redes* partnered with community clinics in those areas and conducted an initial review of 366 medical records of Latina and NHW women who received abnormal screening mammograms, in order to compare the two ethnic groups on the time to obtain a definitive diagnosis of breast cancer.

Inclusion criteria were Latina and NHW women aged >30 with an abnormal screening mammogram. Mammograms were defined as “abnormal” if there were suspicious or incomplete results according to the American College of Radiology (ACR) Breast Imaging and Reporting Data Systems (BI-RADS) (Eberl et al. 2006). Categories included were BI-RADS-0 (“indeterminate”), -3 (“probably benign”), -4 (“suspicious”) and −5 (“highly suggestive of malignancy”).

Medical records were reviewed to obtain age, educational attainment, language preference, dates and results of abnormal breast cancer screening and follow-up diagnostic procedures (e.g. mammogram, MRI, fine-needle aspiration, biopsy), and personal and/or family history of breast cancer. A structured form and a companion computer program were developed in Microsoft Access and distributed to each site to enter the data. The data collection was performed by a trained research staff member, under the supervision of the investigators. This protocol did not deviate from the local standard of care given that it only consisted of data collection. Records were excluded when time to definitive diagnosis (see below) could not be estimated.

Of the 366 original charts reviewed, 30% (n=106) were excluded due to (1) unavailability of mammogram results (n=72), or (2) BI-RADS-1 (“normal;” n=11) or −2 (“benign;” n=23) readings. Only 260 records (186 of Latinas and 74 of NHWs) were retained for analyses after assessment of data quality and completeness.

### Study measures

The main outcome was ‘time to definitive diagnosis,’ calculated as number of days from index abnormal mammogram until definitive diagnosis for breast cancer was reached. ‘Index’ referred to the most recent test performed prior to commencing the diagnostic process (Bevers et al. 2009). Definitive diagnosis was defined as biopsy with pathology report, or clinical determination indicating no further need for evaluation, in accordance with protocol (Freund et al. 2008). Clinical evaluation was included to account for variation in clinical practices. Because of the variability in the number of days to definitive diagnosis, and the likelihood of outliers that would result in skewed data, we censored this outcome at a maximum of 365 days. Other studies of breast cancer diagnosis indicate this exceeds adequate follow-up periods regarding clinical significance (Hershman et al. 2006; National Cancer Institute 2010; Perez-Stable et al. 2012). If diagnosis did not occur within the time frame of the study, cases remained undiagnosed and time was calculated as the interval from their date of index abnormal mammogram to the last known visit date or the end of the study, whichever occurred first. For women with BI-RADS-3 mammogram results and time to diagnosis >180 days, time to definitive diagnosis was shifted backward 180 days to account for the ACR-recommended short-interval follow-up (repeat mammogram in six months) (Aiello Bowles 2010; Battaglia et al. 2010). These data were derived from patient clinical records, scheduling information, and key informant interviews with healthcare personnel from the clinic. Records were excluded from analysis when time to definitive diagnosis could not be calculated from available information, and were compared to included records on all the independent variables for evaluation of potentially induced bias.

The independent variable (ethnicity) was coded as ‘Latina’ and ‘Non-Hispanic white’ using a number of sources as follows: 82% of values for ethnicity were identified directly from a medical chart-specific ethnic note. If not recorded as such, ethnicity was imputed from self-reported national origin, Spanish surname, or primary language given elsewhere in the chart (Eschbach et al. 2005).

Covariate measures included demographic variables: age (30–39, 40–49, 50–59, 60–69, 70+ years), insurance status (Medicaid/Medicare only, Medicare and private, private only, and no insurance), and language use (English only, more English than Spanish, English and Spanish equally, more Spanish than English, Spanish only, and other language spoken). Education level was removed as a covariate due to the preponderance of missing data (80% of the sample). Multiple categories were presented to provide as much detail as possible. Clinical characteristics serving as covariates included personal and family history (FH) of breast cancer (yes/no), breast density (almost entirely fat, scattered fibroglandular densities, and heterogeneous/extremely dense), BI-RADS category, and study site (1–6).

### Statistical analyses

Descriptive group characteristics were used to summarize the data. Chi-square tests for categorical variables and Student t-tests for continuous variables were conducted to assess differences between groups. Median time to diagnosis and its corresponding 95% confidence interval (CI) was used to describe duration of the diagnostic period. Kaplan-Meier estimates for the proportion of undiagnosed women at different times were calculated across each level of the independent variables, and compared using the log-rank test. Time to diagnosis between Latinas and NHWs was then compared using Cox proportional hazards models to obtain adjusted estimates of hazard ratios (aHRs), controlling for significant covariates. Shared-frailty models were used to account for within-site correlation (Duchateau & Janssen 2008; Therneau & Grambsch 2000). Hazard ratios >1.0 are consistent with a shorter time to diagnosis. All analyses were conducted using Stata Version 12.1 (2012, StataCorp LP, College Station, TX).

## Results

To identify factors influencing time to definitive diagnosis, we reviewed clinic records of Latinas and NHWs with abnormal screening mammograms from six U.S. cities participating in the NCI-funded *Redes En Acción* research network. A total of 15 clinics contributed data to the study (data not shown). Clinic size ranged from small practices (5–30 physicians) in suburban or small-to-medium-sized metropolitan areas, to hospitals and medical centers in urban areas (100-500+ bed capacity and 150–1500 physicians). Approximately half the clinics were public non-federal government facilities, and the rest were private non-profits. The percentage of identified Latinas attending these facilities ranged from 10% in Brooklyn, 25% in the Bay Area, 45% in Miami, to >90% at the San Diego and Texas sites.

Table 1 shows sample demographics and clinical characteristics. Data was collected from a total of 260 women, 72% (n=186) of whom were Latina. Mean age was 53.5 years (SD=9.5) for Latinas, significantly younger than NHWs (mean 58.1, SD=10.9, *p<0.001*). Most Latina women had no health insurance (60% vs. 37% of NHWs, *p <0.001*). Among Latinas, significantly more women (33%) spoke predominantly or only Spanish compared to English (16%; *χ*^*2*^*=51.07, p<0.001*; data not shown). The majority of both groups had no personal history (94% and 89% of Latinas and NHWs, respectively), or FH (90% and 72% of Latinas and NHWs, respectively), of breast cancer. Mammography results revealed no significant differences in breast density (*χ*^*2*^*=3.90, p=0.14)* or BI-RADS category (*χ*^*2*^*=2.18, p=0.33)*. Distribution of participants by study site is also shown.

**Table 1:** **Sample demographics and clinical characteristics**

	Latina Women (n=186 )		Non-Hispanic White Women (n=74 )		Total (n=260 )			
	n	%	n	%	n	%	χ^2^ / t	***p***
**Age**							11.79	**0.019**
30 – 39	9	4.8	2	2.7	11	4.2		
40 – 49	57	30.7	17	23.0	74	28.5		
50 – 59	64	34.4	24	32.4	88	33.9		
60 – 69	42	22.6	17	23.0	59	22.7		
70+	9	4.8	13	17.6	22	8.5		
N/A	5	2.7	1	1.4	6	2.3		
Age in years (mean, SD)	53.5	9.5	58.1	10.9	54.8	10.1	−3.38	**<0.001**
**Insurance**							40.94	**<0.001**
Medicaid/Medicare	36	19.4	6	8.1	42	16.2		
Medicare + private	7	3.8	20	27.0	27	10.4		
Private only	31	16.7	21	28.4	52	20.0		
No insurance	112	60.2	27	36.5	139	53.5		
**History of BC**							1.87	0.17
Yes	11	5.9	8	10.8	19	7.3		
No	175	94.1	66	89.2	241	92.7		
**Family History of BC**							14.52	**<0.001**
Yes	18	9.7	21	28.4	39	15.0		
No	168	90.3	53	71.6	221	85.0		
**Breast density**							3.90	0.14
Almost entirely fat	8	4.3	4	5.4	12	4.6		
Scattered fibroglandular densities	42	22.6	20	27.0	62	23.9		
Heterogeneous/extremely dense	62	33.3	14	18.9	76	29.2		
N/A	74	39.8	36	48.7	110	42.3		
**BI-RADS**							2.18	0.33
BI-RADS 0	35	18.8	9	12.2	44	16.9		
BI-RADS 3	35	18.8	18	24.3	53	20.4		
BI-RADS 4/5	116	62.4	47	63.5	163	62.7		
**Study Site**							49.07	**<0.001**
1	20	10.8	25	33.8	45	17.3		
2	34	18.3	*	*	34	13.1		
3	19	10.2	9	12.2	28	10.8		
4	44	23.7	29	39.2	73	28.1		
5	33	17.7	11	14.9	44	16.9		
6	36	19.4	*	*	36	13.9		

**Note:** SD = standard deviation; N/A = not documented in medical record; HS = High School; BC = breast cancer; BI-RADS = Breast Imaging and Reporting Data Systems; * = no patient records reported.

**Figure 1:**
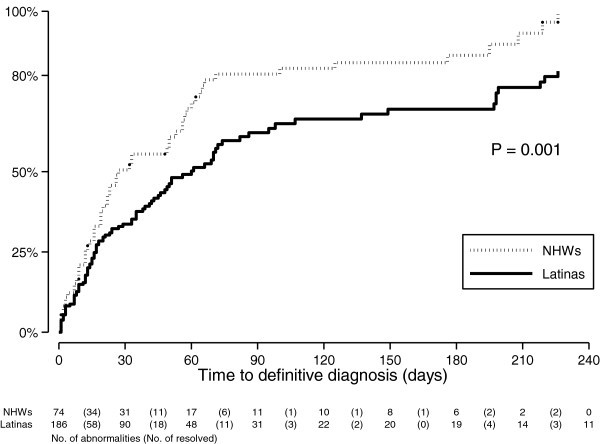
**Kaplan-Meier estimates of the cumulative proportion of women diagnosed by group.** Note: Definitive diagnosis was defined as biopsy with pathology report, or clinical determination indicating no further need for evaluation. Figure is truncated at 240 days, when all NHW mammogram abnormalities were resolved.

Figure 1 shows the Kaplan-Meier estimates of the cumulative proportion of diagnosed women by group at different times. Time for 50% of Latinas to reach a definitive diagnosis was 60 days (SE=8.14; 95% CI 45, 74), significantly longer than NHWs (27 days; SE=5.26; 95% CI 19, 53) (see also Table 2). This gap increased when time for 80% of women to reach diagnosis was calculated, where it took 226 days for Latinas and 71 days for NHWs (χ2=10.32, *p=0.001*).

**Table 2:** **Median time to definitive diagnosis (days since index abnormal mammogram) – By ethnicity, demographics, and clinical characteristics**

	Latina Women (n=186)			Non-Hispanic White Women (n=74)			Total (n=260)			Log-rank test	
	Median	95%	CI	Median	95%	CI	Median	95%	CI	χ^2^	***p***
**Ethnicity**	60	45	74	27	19	53	50	38	60	**10.32**	**0.001**
**Age (years)**										7.53	0.11
30 – 39	33	3	-	12	12	-	20	7	51		
40 – 49	61	35	218	32	3	125	51	32	82		
50 – 59	70	35	149	22	9	50	50	29	71		
60 – 69	60	35	197	58	19	100	60	38	95		
70+	27	7	-	27	7	62	27	16	53		
N/A	-	17	-	-	-	-	195	17	-		
**Insurance**										**8.06**	**0.04**
Medicaid/Medicare	51	24	199	23	9	-	46	24	197		
Medicare + private	218	72	-	60	27	-	64	53	218		
Private only	198	39	260	33	12	58	66	35	198		
No insurance	48	27	70	20	8	32	42	20	56		
**History of BC**										0.08	0.78
Yes	13	5	198	19	3	-	19	11	197		
No	60	45	74	32	20	53	50	39	60		
**Family History of BC**										0.30	0.58
Yes	46	29	260	26	12	62	46	16	64		
No	61	43	74	32	19	56	51	38	65		
**Breast density**										2.73	0.25
Almost entirely fat	13	5	-	23	7	-	18	7	35		
Scattered fibroglandular densities	51	24	86	19	2	32	35	16	56		
Heterogeneous/extremely dense	50	18	199	22	9	-	42	18	61		
N/A	149	66	198	58	23	66	66	56	149		
**BI-RADS Category**										**18.31**	**<0.001**
BI-RADS-0	98	70	-	60	4	-	82	60	107		
BI-RADS-3	95	74	-	66	19	-	125	66	-		
BI-RADS-4/5	38	20	50	26	16	50	33	22	46		

***Note:****N/A = not documented in medical record; HS = High School; BC = breast cancer; - = not enough data to estimate confidence interval; BI-RADS = Breast Imaging and Reporting Data Systems.*

Table 2 shows median time to definitive diagnosis in days by ethnicity, sample demographics, and clinical characteristics. Ethnicity *(χ*^*2*^*=10.32, p=0.001)*, insurance *(χ*^*2*^*=8.06, p=0.04)*, and BI-RADS category (*χ*^*2*^*=18.31, p<0.001)* were associated with time to definitive diagnosis in the log-rank test. There was no association based on age category, personal or FH of breast cancer, or abnormal breast density on mammography.

Table 3 shows adjusted hazard ratios (aHR) for Cox proportional hazards models of median time to definitive diagnosis. Only covariates that were significantly different by log-rank test (Table 2) were included in the models. When adjusted by site only, ethnicity and more severe BI-RADS category were significantly associated with time to definitive diagnosis. NHWs had a 49% higher rate of definitive diagnosis of breast cancer than Latinas (95% CI 1.03, 2.13; *p=0.035*), and women with BI-RADS-4/5 mammograms likewise were diagnosed at a higher rate than BI-RADS-3 (aHR = 1.95; 95% CI 1.10, 3.49; *p=0.*023). After adjusting for both site and BI-RADS, the rate of diagnosis for NHWs remained significantly higher than for Latinas (aHR=1.59; 95% CI 1.09, 2.31; *p=0.015*). BI-RADS-4/5 also led to a higher rate of definitive diagnosis in the multivariate model (aHR = 2.11; 95% CI 1.18, 3.78; *p=0.011)*. Insurance status did not influence time to definitive diagnosis.

**Table 3:** **Factors associated with time to definitive diagnosis of breast cancer**

	Site-adjusted hazard ratios				Multivariate-adjusted model*			
	aHR	95%	CI	***p***	aHR	95%	CI	***p***
**Ethnicity**								
Latina	1.00	Ref.			1.00	Ref.		
Non-Hispanic White	1.49	1.03	2.13	***0.035***	1.59	1.09	2.31	***0.015***
**Insurance**								
Medicaid/Medicare	1.04	0.65	1.65	*0.873*				
Medicare + private	0.98	0.47	2.06	*0.961*				
Private only	0.92	0.56	1.51	*0.737*				
No insurance	1.00	Ref.						
**BI-RADS**								
BI-RADS 0	1.60	0.74	3.45	*0.231*	1.80	0.83	3.90	*0.137*
BI-RADS 3	1.00	Ref.			1.00	Ref.		
BI-RADS 4/5	1.95	1.10	3.49	***0.023***	2.11	1.18	3.78	***0.011***

***Note:***aHR = adjusted Hazard Ratio; Ref. = reference category; BI-RADS = Breast Imaging and Reporting Data Systems. * = adjusted for site and BI-RADS category.

## Discussion

Our study results indicate that Latina ethnicity plays a significant role in delaying median time to diagnosis of breast cancer in a multi-site sample. This supersedes age, education, insurance status, personal or family history of breast cancer, and mammographic breast density abnormalities. In contrast, results from the CDC’s National Breast and Cervical Cancer Early Detection Program (NBCCEDP) (Caplan et al. 2000) showed diagnosis occurring in <60 days for a majority of women regardless of ethnicity. One possible reason for this difference is that none of our clinic sites participated in the CDC program. Another explanation could be the relatively late arrival of Latina-focused breast cancer research. Most disparity studies in the past six years have compared white and African American populations (Gorin et al. 2006; Blackman & Masi 2006; Newman & Martin 2007), and the ACS’s latest Facts and Figures publication likewise focused on these two groups (American Cancer Society 2011c). In a recent study by one of our collaborators, in which Latinas comprised 18% of women surveyed, 84% of BI-RADS-4/5 women were diagnosed by the recommended 60-day limit. Women with BI-RADS-3 results took significantly longer (183 days vs. 29 for BI-RADS-4/5) to reach diagnosis or resolution as defined by the authors (Perez-Stable et al. 2012). This is consistent with our own findings in this study and elsewhere (Ramirez et al. 2012).

Time-to-event analyses have been done to examine breast cancer recurrence mediated by cancer subtype (Buist et al. 2010), effects of patient navigation (PN) on time to diagnostic resolution in breast and cervical cancer (Markossian et al. 2012), and surgical risk reduction in women with familial ovarian cancer (Manchanda et al. 2012). To our knowledge, this is the first study using time-to-event analysis to examine the disparity in time to definitive diagnosis of breast cancer between Latinas and NHWs. Our data showed an increasing time lag for Latinas relative to NHWs, from twice as long for 50% of Latinas to be diagnosed, to three times to achieve 80% diagnosed.

Limitations of our study include its retrospective design and dependence on medical records, resulting in the unavailability (N/A) of specific data (e.g., educational attainment – see Table 1) across study sites. Thus, site-by-site comparisons could not be made; instead, a pooled analysis was done to interpret available data. This also contributed to the imbalance in total number of eligible Latina and NHW records examined across sites (*χ*^*2*^*=49.07, p<0.001*), which likely accounts for the significant differences observed in 50% of group characteristics. Moreover, our conclusions are limited to Latinas and NHWs, to the exclusion of other minority populations (e.g., African-Americans), in whom the substantial challenge to decrease breast cancer incidence, morbidity and mortality remains (Blackman & Masi 2006; Fedewa et al. 2011; 2012b).

Studies have repeatedly shown that cancer places an unequal burden among women who are of lower socioeconomic status and/or ethnic minorities (Jones et al. 2005; Caplan et al. 2000; Chang et al. 1996; Kerlikowske 1996). These disparities manifest themselves in lower survival rates and have been shown to result from a cluster of circumstances, including minority status and marginalization, inability to access and adequately utilize medical resources, unavailability of those resources in some locales, late diagnoses and more severe disease, and similar delays in treatment ultimately leading to higher rates of death (Eberl et al. 2006; Peres 2010; Karliner & Kerlikowske 2007). Our study highlights the significance of ethnicity in delaying breast cancer diagnosis among Latinas, who represent a minority group considered the youngest and fastest-growing in the U.S. (U.S. Census Bureau, American Community Survey 2006). Our results underscore the clear need for increased Latina-targeted breast cancer prevention and screening services as part of a multi-level approach to cancer care delivery (Taplin et al. 2012) that ultimately improves quality of care by improving outcomes.

One of the Healthy People 2020 objectives is to increase breast cancer screening by 10% from the current 73.7% of females who receive guideline-based screening (Centers for Disease Control and Prevention and National Center for Health Statistics 2012a). However, for Latinas the current age-adjusted rate is only 68.3% (Centers for Disease Control and Prevention and National Center for Health Statistics 2012b), thus necessitating even greater efforts to reach this goal. Moreover, with cancer now the leading cause of death among Latinos (Siegel et al. 2012), research and intervention efforts need to expand to decrease lag time between index abnormal mammogram and definitive diagnosis. Suggested interventions to ameliorate disparities have included counseling, health education, and PN (Blackman & Masi 2006). Such interventions, applied correctly and in a timely fashion to specific target populations (Ramirez et al. 2012) and clinical challenges (Freeman 2012; Fiscella et al. 2012; Battaglia et al. 2012; Paskett et al. 2012) should streamline the continuum of cancer care from screening through survivorship.

## References

[CR1_144] National Healthcare Disparities Report, 2007 2008 Rockville, MD Agency for Healthcare Research and Quality

[CR2_144] Aiello Bowles EJ Recommendation for short-interval follow-up examinations after a probably benign assessment: is clinical practice consistent with BI-RADS guidance? AJR Am J Roentgenol 2010 194 1152 1159 10.2214/AJR.09.3064 20308525 PMC2861652

[CR3_144] Cancer Facts and Figures for Hispanics/Latinos 2009–2011 2009

[CR4_144] Cancer Prevention and Early Detection Facts and Figures 2011 2011a

[CR5_144] Breast Cancer Facts and Figures 2011–2012 2011b

[CR6_144] Breast Cancer Facts and Figures 2011 Atlanta, GA American Cancer Society, Inc

[CR7_144] Battaglia TA Santana MC Bak S Predictors of timely follow-Up after abnormal cancer screening among women seeking care at urban community health centers Cancer 2010 116 913 921 10.1002/cncr.24851 20052731 PMC2819638

[CR8_144] Battaglia TA Bak SM Heeren T Boston patient navigation research program: the impact of navigation on time to diagnostic resolution after abnormal cancer screening Cancer Epidemiol Biomarkers Prev 2012 21 10 1645 54 10.1158/1055-9965.EPI-12-0532 23045539 PMC3472624

[CR9_144] Bevers TB Anderson BO Bonaccio E NCCN clinical practice guidelines in oncology: breast cancer screening and diagnosis J Natl Compr Canc Netw 2009 7 10 1060 96 19930975 10.6004/jnccn.2009.0070

[CR10_144] Blackman DJ Masi CM Racial and ethnic disparities in breast cancer mortality: are we doing enough to address the root causes? J Clin Oncol 2006 24 14 2170 8 10.1200/JCO.2005.05.4734 16682736

[CR11_144] Buist DS Abraham LA Barlow WE Diagnosis of second breast cancer events after initial diagnosis of early stage breast cancer Breast Cancer Res Treat 2010 124 3 863 73 10.1007/s10549-010-1106-6 20700648 PMC2976764

[CR12_144] Caplan LS May DS Richardson LC Time to diagnosis and treatment of breast cancer: results from the national breast and cervical cancer early detection program, 1991–1995 Am J Public Health 2000 90 1 130 4 10.2105/AJPH.90.1.130 10630153 PMC1446126

[CR13_144] Healthy People 2020 Topics and Objectives: Cancer Objective C-17. 2010 [cited 2012 23 October]; 2nd:[Increase the proportion of women who receive a breast cancer screening based on the most recent guidelines] 2012

[CR14_144] National Health Interview Survey: Breast cancer screening (percent). HP 2020 2008 [cited 2012 23 October]; Percent of women who receive a breast cancer screening based on the most recent guidelines, percent] 2012

[CR15_144] Chang SW Kerlikowske K Napoles-Springer A Racial differences in timeliness of follow-up after abnormal screening mammography Cancer 1996 78 1395 1402 10.1002/(SICI)1097-0142(19961001)78:7<1395::AID-CNCR5>3.0.CO;2-K 8839544

[CR16_144] Cronin KA Richardson LC Henley SJ Vital signs: racial disparities in breast cancer severity - United States, 2005-2009 MMWR Morb Mortal Wkly Rep 2012 61 922 926 23151952

[CR17_144] Duchateau L Janssen P The frailty model. Statistics for biology and health 2008 New York, NY Springer 336

[CR18_144] Eberl MM Fox CH Edge SB BI-RADS classification for management of abnormal mammograms J Am Board Fam Med 2006 19 2 161 164 10.3122/jabfm.19.2.161 16513904

[CR19_144] Ell K Vourlekis B Lee PJ Patient navigation and case management following an abnormal mammogram: a randomized clinical trial Prev Med 2007 44 1 p. 26 33 10.1016/j.ypmed.2006.08.001 16962652

[CR20_144] Elmore JG Armstrong K Lehman CD Screening for breast cancer JAMA 2005 293 10 1245 56 10.1001/jama.293.10.1245 15755947 PMC3149836

[CR21_144] Eschbach K Mahnken JD Goodwin JS Neighborhood composition and incidence of cancer among Hispanics in the United States Cancer 2005 103 5 1036 44 10.1002/cncr.20885 15672387 PMC1853250

[CR22_144] Fedewa SA Edge SB Stewart AK Race and ethnicity are associated with delays in breast cancer treatment (2003–2006) J Health Care Poor Underserved 2011 22 1 128 41 21317511 10.1353/hpu.2011.0006

[CR23_144] Feig SA Screening mammography controversies: resolved, partly resolved, and unresolved Breast J 2005 11 Suppl 1 S3 6 10.1111/j.1075-122X.2005.217161.x 15725113

[CR24_144] Fiscella K Whitley E Hendren S Patient navigation for breast and colorectal cancer treatment: a randomized trial Cancer Epidemiol Biomarkers Prev 2012 21 10 1673 81 10.1158/1055-9965.EPI-12-0506 23045542 PMC3724524

[CR25_144] Freeman HP The origin, evolution, and principles of patient navigation Cancer Epidemiol Biomarkers Prev 2012 21 10 1614 7 10.1158/1055-9965.EPI-12-0982 23045534

[CR26_144] Freund KM Battaglia TA Calhoun E National cancer institute patient navigation research program: methods, protocol, and measures Cancer 2008 113 12 3391 9 10.1002/cncr.23960 18951521 PMC2698219

[CR27_144] Gorin SS Heck JE Cheng B Delays in breast cancer diagnosis and treatment by racial/ethnic group Arch Intern Med 2006 166 20 p. 2244 52 10.1001/archinte.166.20.2244 17101943

[CR28_144] Green BB Taplin SH Breast cancer screening controversies J Am Board Fam Pract 2003 16 3 233 41 10.3122/jabfm.16.3.233 12755251

[CR29_144] Guerra CE Krumholz M Shea JA Literacy and knowledge, attitudes and behavior about mammography in Latinas J Health Care Poor Underserved 2005 16 1 152 66 10.1353/hpu.2005.0012 15741716

[CR30_144] Heckman BD Fisher EB Monsees B Coping and anxiety in women recalled for additional diagnostic procedures following an abnormal screening mammogram Health Psychol 2004 23 1 42 8 10.1037/0278-6133.23.1.42 14756602

[CR31_144] Hershman DL Wang X McBride R Delay in initiating adjuvant radiotherapy following breast conservation surgery and its impact on survival Int J Radiat Oncol Biol Physiol 2006 65 1353 1360 10.1016/j.ijrobp.2006.03.048 16765531

[CR32_144] Howe HL Wu X Ries LAG Annual report to the nation on the status of cancer, 1975–2003, featuring cancer among US Hispanic/Latino populations Cancer 2006 107 8 1711 1742 10.1002/cncr.22193 16958083

[CR33_144] Jones BA Dailey A Calvocoressi L Inadequate follow-up of abnormal screening mammograms: findings from the race differences in screening mammography process study (United States) Cancer Causes Control 2005 16 809 821 10.1007/s10552-005-2905-7 16132791

[CR34_144] Kaplan C Eisenberg M Erickson P Barriers to breast abnormality follow-up: minority, low-income patients’ and their providers’ view Ethn Dis 2005 15 720 726 16259499

[CR35_144] Karliner LS Kerlikowske K Ethnic disparities in breast cancer Womens Health (Lond Engl) 2007 3 6 679 88 10.2217/17455057.3.6.679 19803977

[CR36_144] Kerlikowske K Timeliness of follow-up after abnormal screening mammography Breast Cancer Res Treat 1996 40 1 53 64 10.1007/BF01806002 8888152

[CR37_144] Klabunde CN Brown M Ballard-Barbash R Cancer screening - United States, 2010 Morb Mortal Wkly Rep 2012 61 41 45 22278157

[CR38_144] Knutson D Steiner E Screening for breast cancer: current recommendations and future directions Am Fam Physician 2007 75 11 1660 6 17575656

[CR39_144] Manchanda R Burnell M Abdelraheim A Factors influencing uptake and timing of risk reducing salpingo-oophorectomy in women at risk of familial ovarian cancer: a competing risk time to event analysis BJOG 2012 119 5 527 36 10.1111/j.1471-0528.2011.03257.x 22260402

[CR40_144] Markossian TW Darnell JS Calhoun EA Follow-up and timeliness after an abnormal cancer screening among underserved, urban women in a patient navigation program Cancer Epidemiol Biomarkers Prev 2012 21 10 1691 700 10.1158/1055-9965.EPI-12-0535 23045544 PMC3482137

[CR41_144] Miller JW, King JB, Joseph DA: **Breast cancer screening among adult women--behavioral risk factor surveillance system, united states, 2010.** *MMWR Morb Mortal Wkly Rep* 2012, (61 Suppl)**:**p. 46–50. 22695463

[CR42_144] Cancer Trends Progress Report - 2009/2010 Update. 2010 April 2010

[CR43_144] Newman LA Martin IK Disparities in breast cancer Curr Probl Cancer 2007 31 3 134 56 10.1016/j.currproblcancer.2007.01.003 17543945

[CR44_144] Paskett ED Katz ML Post DM The ohio patient navigation research program: does the american cancer society patient navigation model improve time to resolution in patients with abnormal screening tests? Cancer Epidemiol Biomarkers Prev 2012 21 10 1620 1628 10.1158/1055-9965.EPI-12-0523 23045536 PMC3785236

[CR45_144] National Cancer Institute: PDQ® Breast Cancer Screening. PDQ® Cancer Information Summaries 2012

[CR46_144] Peres J Mammography screening: after the storm, calls for more personalized approaches J Natl Cancer Inst 2010 102 1 9 11 10.1093/jnci/djp496 20023204

[CR47_144] Perez-Stable E Afable-Munsuz A Kaplan CP Factors influencing time to diagnosis after abnormal mammography in diverse women J Womens Health 2013 22 2 159 166 10.1089/jwh.2012.3646 PMC3573728 23350859

[CR48_144] Ramirez AG Talavera GA Villarreal R Breast cancer screening in regional hispanic populations Health Educ Res 2000 15 5 559 68 10.1093/her/15.5.559 11184215

[CR49_144] Ramirez AG Perez-Stable EJ Talavera GA Navigating latinas with breast screen abnormalities to diagnosis: the Six cities study Cancer 2012 10.1002/cncr.27912 PMC3628781 23233265

[CR50_144] Shavers VL Brown ML Racial and ethnic disparities in the receipt of cancer treatment J Natl Cancer Inst 2002 94 5 334 57 10.1093/jnci/94.5.334 11880473

[CR51_144] Siegel R, Naishadham D, Jemal A: **Cancer statistics for hispanics/latinos, 2012.** *CA Cancer J Clin* 2012.,**62**(5)**:**

[CR52_144] Steinberg ML Fremont A Khan DC Lay patient navigator program implementation for equal access to cancer care and clinical trials: essential steps and initial challenges Cancer 2006 107 11 2669 77 10.1002/cncr.22319 17078056

[CR53_144] Taplin SH Yabroff KR Zapka J A multilevel research perspective on cancer care delivery: the example of follow-up to an abnormal mammogram Cancer Epidemiol Biomarkers Prev 2012 21 10 1709 15 10.1158/1055-9965.EPI-12-0265 22911332 PMC3467321

[CR54_144] Therneau TM Grambsch PM Modeling survival data: extending the Cox model. 1st ed. Statistics for Biology and Health 2000 New York, NY Springer

[CR55_144] U.S. Interim Projections by Age, Sex, Race, and Hispanic Origin: Table 1a Projected Population of the United States, by Race and Hispanic Origin – 2000 to 2050 2004

[CR56_144] American Community Survey: Selected population profile in the United States: Hispanic or Latino 2011

[CR57_144] U.S. Preventive Services Task Force Screening for breast cancer: recommendations and rationale Ann Intern Med 2002 137 5 Part 1 p. 344 6 10.7326/0003-4819-137-5_part_1-200209030-00011 12204019

[CR58_144] U.S. Preventive Services Task Force, Screening for breast cancer U.S. Preventive services task force recommendation statement Ann Intern Med 2009 151 p. 716 726 10.7326/0003-4819-151-10-200911170-00008 19920272

[CR59_144] Vahabi M Breast cancer screening methods: a review of the evidence Health Care Women Int 2003 24 9 773 93 10.1080/07399330390229957 14742116

